# Deep Learning-Based Pain Classifier Based on the Facial Expression in Critically Ill Patients

**DOI:** 10.3389/fmed.2022.851690

**Published:** 2022-03-17

**Authors:** Chieh-Liang Wu, Shu-Fang Liu, Tian-Li Yu, Sou-Jen Shih, Chih-Hung Chang, Shih-Fang Yang Mao, Yueh-Se Li, Hui-Jiun Chen, Chia-Chen Chen, Wen-Cheng Chao

**Affiliations:** ^1^Department of Critical Care Medicine, Taichung Veterans General Hospital, Taichung, Taiwan; ^2^Department of Industrial Engineering and Enterprise Information, Tunghai University, Taichung, Taiwan; ^3^Artificial Intelligence Studio, Taichung Veterans General Hospital, Taichung, Taiwan; ^4^Colledge of Medicine, National Chung Hsing University, Taichung, Taiwan; ^5^Department of Nursing, Taichung Veterans General Hospital, Taichung, Taiwan; ^6^Department of Electrical Engineering, National Taiwan University, Taipei, Taiwan; ^7^Electronic and Optoelectronic System Research Laboratories, Industrial Technology Research Institute, Hsinchu, Taiwan; ^8^Department of Automatic Control Engineering, Feng Chia University, Taichung, Taiwan; ^9^Big Data Center, National Chung Hsing University, Taichung, Taiwan

**Keywords:** pain, critically ill patients, facial expression, artificial intelligence, classifier

## Abstract

**Objective:**

Pain assessment based on facial expressions is an essential issue in critically ill patients, but an automated assessment tool is still lacking. We conducted this prospective study to establish the deep learning-based pain classifier based on facial expressions.

**Methods:**

We enrolled critically ill patients during 2020–2021 at a tertiary hospital in central Taiwan and recorded video clips with labeled pain scores based on facial expressions, such as relaxed (0), tense (1), and grimacing (2). We established both image- and video-based pain classifiers through using convolutional neural network (CNN) models, such as Resnet34, VGG16, and InceptionV1 and bidirectional long short-term memory networks (BiLSTM). The performance of classifiers in the test dataset was determined by accuracy, sensitivity, and F1-score.

**Results:**

A total of 63 participants with 746 video clips were eligible for analysis. The accuracy of using Resnet34 in the polychromous image-based classifier for pain scores 0, 1, 2 was merely 0.5589, and the accuracy of dichotomous pain classifiers between 0 vs. 1/2 and 0 vs. 2 were 0.7668 and 0.8593, respectively. Similar accuracy of image-based pain classifier was found using VGG16 and InceptionV1. The accuracy of the video-based pain classifier to classify 0 vs. 1/2 and 0 vs. 2 was approximately 0.81 and 0.88, respectively. We further tested the performance of established classifiers without reference, mimicking clinical scenarios with a new patient, and found the performance remained high.

**Conclusions:**

The present study demonstrates the practical application of deep learning-based automated pain assessment in critically ill patients, and more studies are warranted to validate our findings.

## Background

Pain is an essential medical issue but somehow difficult to assess in critically ill patients who cannot report their pain (1). Therefore, the Critical-Care Pain Observation Tool (CPOT) has been developed to grade the pain through assessing behavior alternations, such as facial expressions, among critically ill patients in the past two decades (2). The facial expression is the fundamental behavior alternation in CPOT and consists of relaxed, tense, and grimacing (pain score 0, 1, and 2) (3). Currently, facial expression-based pain assessment is graded by the nurse, and there is an unmet need to develop an automated pain assessment tool based on facial expression to relieve the medical staff from the aforementioned workload (4).

A number of automated recognition of facial expressions of pain and emotion has been developed through using distinct approaches (5–9). Pedersen et al. used Support Vector Machine (SVM) as a facial expression-based pain classifier in UNBC-McMaster Shoulder Pain Expression Archive Database, consisting of 200 video sequences obtained from 25 patients with shoulder pain, and reported that the accuracy of the leave-one-subject-out 25-fold cross was 0.861 (7). Given that video sequences contain temporal information with respect to pain, two studies were used Recurrent Neural Network (RNN) and hybrid network to extract the time-frame feature among images and reported an improved performance (8, 9). Furthermore, recent studies have employed fusion network architectures and further improved the F1 score to ~0.94 (10, 11). Therefore, the recent advancements in deep learning might enable us to establish a facial expressed-based pain assessment tool in critically ill patients.

Notably, the application of the aforementioned methods in critically ill patients might not be straightforward due to real-world difficulties to obtain standardized and whole unmasked facial images of patients admitted to the intensive care unit (ICU) (12). Unlike the high-quality whole facial image in the UNBC-McMaster Shoulder Pain Expression Archive Database, critically ill patients may have masks on the face due to needed medical devices, such as endotracheal tube, nasoesophageal tube, and oxygen mask. Furthermore, pain-associated facial muscle movements might hence be subtle due to sedation and tissue oedema in critically ill patients. Therefore, there is a substantial need for using facial images obtained in sub-optimal real-world conditions at ICUs to establish an automated facial expression-based assessment tool for pain in critically ill patients. In the present prospective study, we recorded facial video clips in critically ill patients at the ICUs of Taichung Veterans General Hospital (TCVGH) and employed an ensemble of three Convolutional Neural Network (CNN) models as well as RNN to establish the pain classifier based on facial expressions.

## Materials and Methods

### Ethical Approval

This study was approved by the Institutional Review Board approval of the Taichung Veterans General Hospital (CE20325A). Informed consent was obtained from all of the participants prior to the enrollment in the study and collection of data.

### Study Population

We conducted this prospective study by enrolling patients who were admitted to medical and surgical ICUs at TCVGH, a referral hospital with 1,560 beds in central Taiwan, between 2020-Nov and 2021-Nov. The CPOT is a standard of care in the study hospital, and grading of the facial expression-based pain score is in accordance with the guideline (3). In detail, a score of 0 is given if there is no observed muscle tension in the face, and the score of 1 is composed of a tensed muscle contraction, such as the presence of frowning, brow lowering, orbit tightening as well as levator muscle contraction. The score of 2 consists of grimacing, which is a contraction of facial muscles, particularly muscles nearby the eyebrow area, plus eyelid tightly closed.

### Video Sequences With Labeled Pain Grade Based on Facial Expression

**Figure 1:**
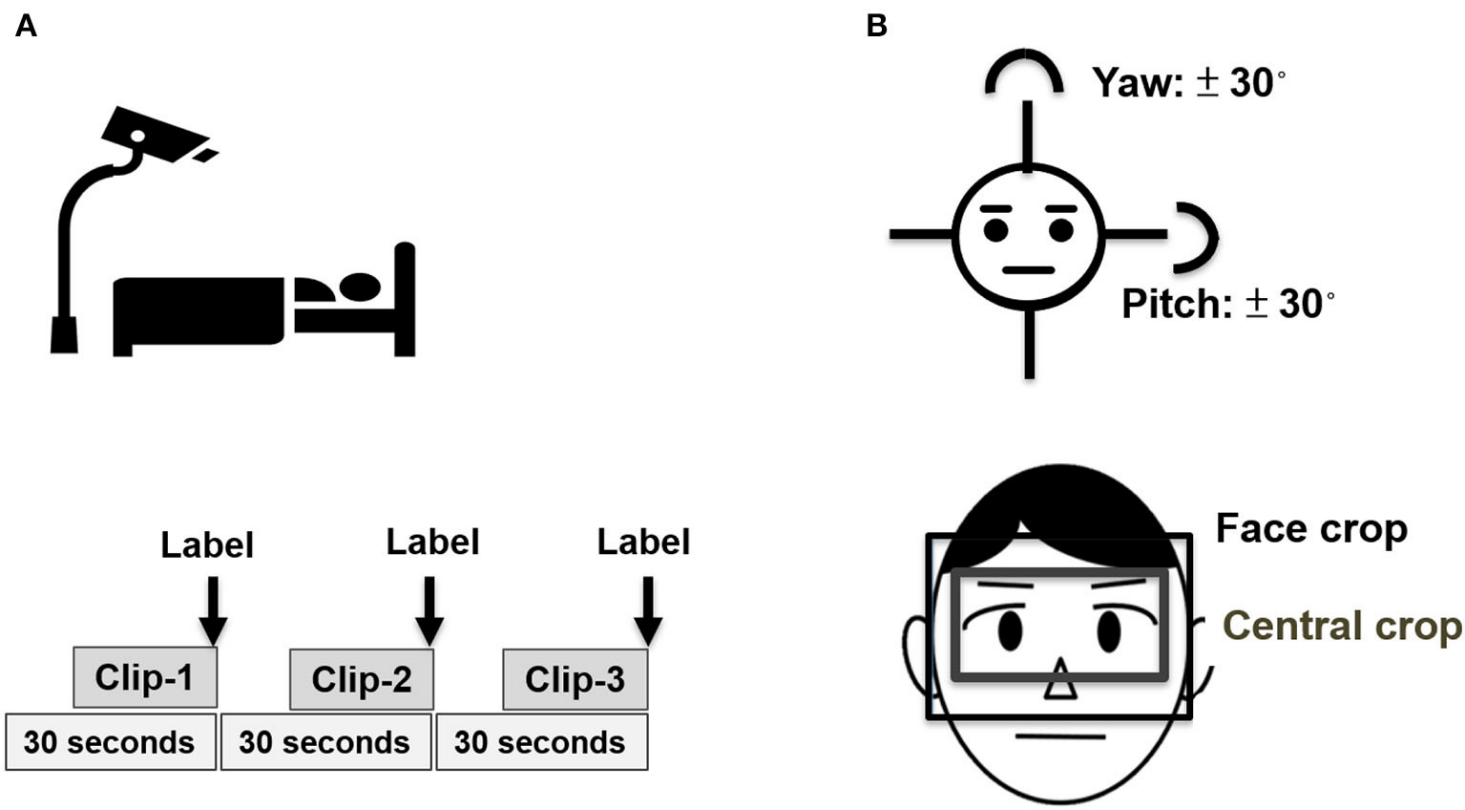
Schematic diagram of image acquisition and preprocessing. **(A)** Recording of video clips with labeling and **(B)** Preprocessing of video sequences.

Figure 1 depicts the protocol of video record, labeling, and image preprocessing of the present study (Figure 1). Video record and labeling were performed by three experienced nurses after training for inter-rater concordance, and the labeling was further validated by two senior registered nurses. To mitigate information bias and synchronize the recording and labeling, we designed a user interface that enables the study nurse to observe the patient for 10 s, to record a video for 20 s, and then label the pain score at the end of the video. To further reduce the potential sampling errors, we recorded three labeled videos in each observation; therefore, each 90-s video sequence has three 20-s clips (Figure 1A). Given the nature of observation of this study, we conducted the recording per day during the ICU admission of participants, particularly before and after suction, dressing change as well as invasive procedures, to obtain the videos with distinct pain grades in individual critically ill patients. With regards to the hardware, the frame per second of the applied camera was 30, and the total frames of a 20-s video clip were nearly 400–600 frames per clip. To standardize the video clips, we used 50 frames in each 20-s clip; therefore, there were 2.5 representative frames per second for the following experiments. To avoid any interference with critical care, we designed a portable camera rack that enables us to take high-quality video ~1–2 m from the patient.

### Image Preprocessing

We used a facial landmark tracker to locate the facial area (13). Due to the face that was masked by the aforementioned medical devices might not be detected by the facial landmark tracker, we further used multi-task CNN to locate the facial area if the face was not located by the facial landmark tracker (14). Given that the area nearby the eyebrow is the key area to interpret pain score, we hence cropped the face between hairline and nose not only to focus on the eyebrow area, but also to avoid the confounding of the aforementioned medical devices. We further cropped the central part of the eyebrow area with a fixed ratio of height/width (3/4) for the following experiments. Given that facial images with extreme angles may lead to the facial landmark misalignment and affect the following experiments, we hence excluded the faces with yaw or pitch angle over 30 degrees (Figure 1B).

### Image-Based Pain Classifiers

**Figure 2:**
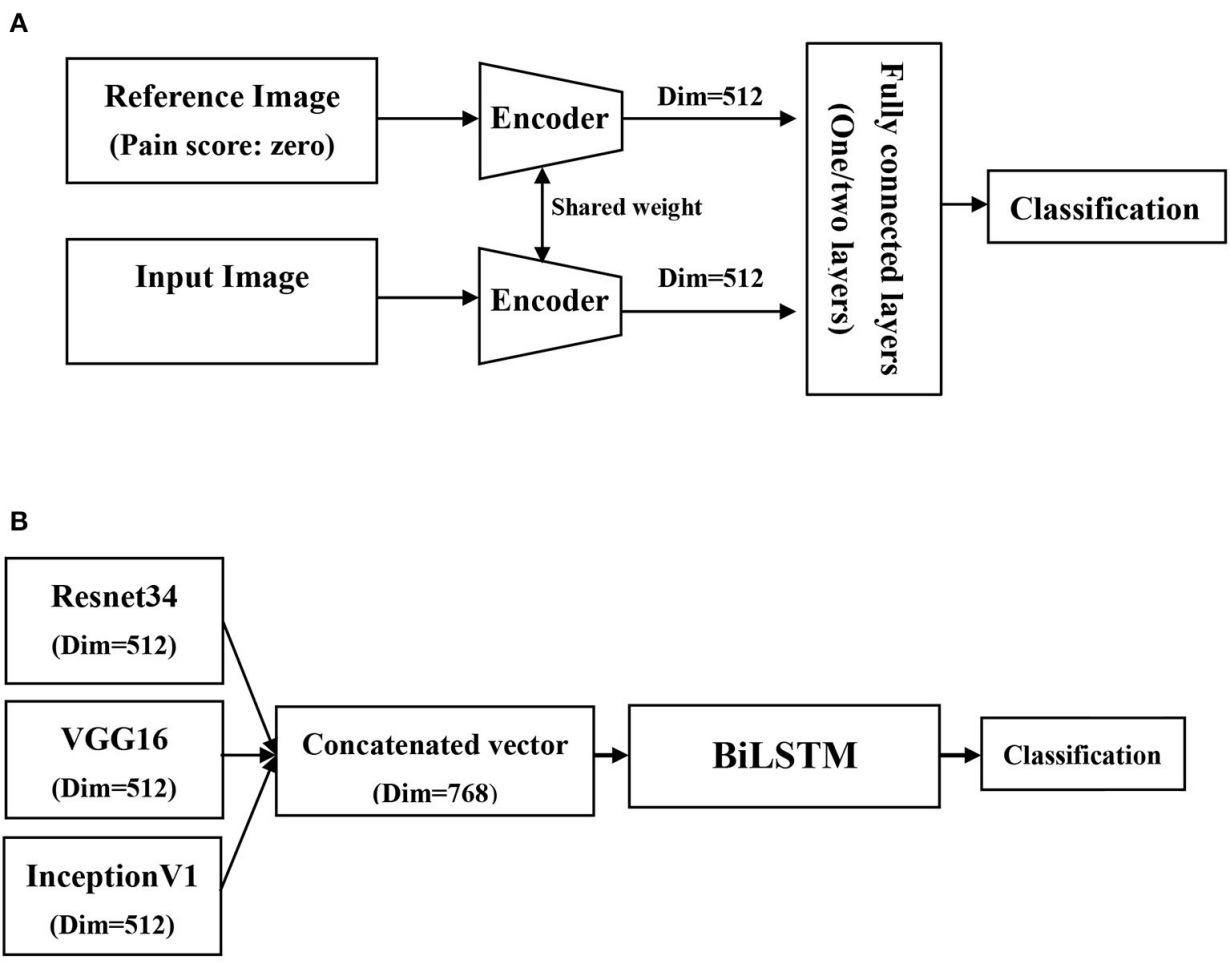
Schematic diagram of network architectures in the present study. **(A)** Image-based pain classifiers using relation and siamese network architecture, **(B)** Video-base pain classifier using bidirectional long short-term memory networks (BiLSTM).

Figure 2 illustrates the deep learning-based Siamese network architectures for image- and video-based pain classifiers in this study (15) (Figure 2). To reduce the need for an extremely high number of labeled but unrelated images for learning, we employed a relation network architecture for the image-based pain classifier (16). In brief, the aforementioned relation network is designed for learning to compare the differences among labeled images of each individual patient; therefore, the essential need is the images with distinct grades among individual patients, instead of a high number of unrelated images from patients with high heterogeneity. Therefore, we used the data of the 63 participants who had images of all of 0, 1, 2 labeled images. In detail, by feeding grade-0 facial expression image and grade 1/2 images into CNN encoder, two vectors were obtained to represent the subtle difference between the image of grade-0 and grade-1/2, instead of calculating the complex distance metric of two images in high dimensions. Indeed, the application of relation network should be in line with clinical grading of pain by the nurse, who had to recognize the baseline facial appearance of an individual patient prior to grade pain-score based on the facial expression. In this study, we used three CNN models that have fewer vanishing gradient issues, such as Resnet34, VGG16, and inceptionV1, as well as two types of the fully connected layer set up with one and two layers (17–19). Therefore, there were a total of six combinations for the image-based pain classifier, and we applied the voting to optimize the classifier performance through averaging outputs of different models. With regards to the main hyperparameters, we used the cross-entropy loss as the loss function in the image-based pain classifier, and the learning rate, optimizer, and trained epochs were 1e-4, Adam, and 60 epochs, respectively.

### Video-Based Pain Classifiers

With regard to the video-based pain classifier, we employed a many-to-one sequence model given that the output of this study is a one pain grade. Similar to the image-based pain classifier, we used a Siamese network architecture as feature extractors. Given that multiple CNN encoders were used in the present study, we hence processed the image through three CNN encoders to get three vectors and concatenate these vectors to a relatively low-dimensional space. The concatenated output vectors of each frame were then fed into the bidirectional long short-term memory networks (BiLSTM) for the classification of pain (20). Given that the CNN encoder had been trained in the image-based pain classifier, we hence reduced the learning rate to 1e-5 on the video-based pain classifier and froze the weights of the CNN encoder in the first 10 epochs, and this approach may facilitate to focus on training BiLSTM in the first 10 epochs. The other parameters, such as loss function, optimizer, and trained epochs, were in line with those used in the image-based pain classifier.

### Statistical Analyses

Data were expressed in frequency of occurrence (percentages) for categorical variables and as means ± *SD* for continuous variables. Differences between the survivor and non-survivor groups were analyzed using Student's *t*-test for continuous variables and Fisher's exact test for categorical variables. The proportion of train, validation, and test datasets were 60, 20, and 20%, respectively. The performance of the pain classifier in the test dataset was determined by accuracy, sensitivity, and F1-score. Python version 3.8, PyTorch 1.9.1, and CUDA 11.1 were used in this study.

## Results

### Patients' Characteristics

A total of 341 participants were enrolled, and there were 7,813 qualified videos, of which the number of scores 0, 1, and 2 were 5,717, 1,714, and 382, respectively. Given that we employed relation network architecture in this study, we hence used images among 63 participants who had all of the pain-score 0, 1, and 2 labeled video clips, and the number of videos with 0, 1, and 2 were 351, 253, and 142, respectively. The mean age of included patients for analyses was 69.3 ± 14.6 years, and 55.6 (35/63) of them was male (Table 1). The majority (81.0%, 51/63) of enrolled participants were critically ill patients who were admitted to medical ICUs. The ICU severity scores of acute physiology and chronic health evaluation II (APACHE II), sequential organ failure assessment (SOFA) day-1, SOFA day-3, and SOFA day-7 were 25.3 ± 5.7, 9.0 ± 3.7, 8.5 ± 4.1, and 8.2 ± 3.8, respectively.

**Table 1:** Characteristics of the enrolled 63 participants who had videos with all of three pain-score categories.

**Basic data**	
Age, years	69.3 ± 14.6
Sex (male)	35 (55.6%)
Height (cm)	160.1 ± 8.0
Body weight (kgs)	57.3 ± 10.0
**ICU types**	
Medical ICUs	51 (81.0%)
Surgical ICUs	12 (19.0%)
**Laboratory data (Day-1)**	
White blood cell counts (/ml)	13,670.7 ± 11,259.5
Hematocrit (%)	28.8 ± 8.4
Creatinine (mg/dl)	1.9 ± 1.4
Sodium (mg/dl)	140.3 ± 5.5
Potassium (mg/dl)	4.0 ± 0.7
**Severity scores**	
APACHE II score	25.3 ± 5.7
SOFA score, day-1	9.0 ± 3.7
SOFA score, day-3	8.5 ± 4.1
SOFA score, day-7	8.2 ± 3.8

*Data were presented as mean ± standard deviation and number (percentage). ICU, intensive care unit; APACHE II, acute physiology and chronic health evaluation II; SOFA, sequential organ failure assessment*.

### Performance of Image-Based Pain Classifiers

In image-based pain classifiers, we attempted to classify with three pain categories (0, 1, and 2) and dichotomous pain classifiers (0 vs. 1/2 and 0 vs. 2) given pain score = 2 reflects a clinical warning signaling requiring immediate clinical evaluation and management (Table 2). In Resnet34 with one fully connected layer (1024, 3), the performance of the polychromous classifier for 0, 1, and 2 appeared to be suboptimal, with the accuracy, sensitivity, and F1 score were merely 0.5589, 0.5589, and 0.5495, respectively. The performance of the two dichotomous image-based pain classifiers was much higher than that in polychromous pain classifier. The accuracy, sensitivity, and F1 score were 0.7668, 0.8422, and 0.8593 to classify 0 vs. 1/2 and were 0.8593, 0.8925, and 0.8638 to classify 0 vs. 2. We further tested the performance of using VGG16, InceptionV1, and two fully connected layers. The performances of Resnet34 and VGG16 were slightly higher than that of InceptionV1. For example, the accuracy of dichotomous pain classifier between 0 vs. 1/2 in Resnet34, VGG16, and Inception were 0.7668, 0.7578, and 0.7055, respectively. With regard to the efficacy of using two fully connected layers ([1024, 256] followed by [256, 3]), the performance tended to improve in a few models, such as dichotomous pain classifier between 0 vs. 1/2 in InceptionV1 (accuracy increased from 0.7055 to 0.7587).

**Table 2:** Performance image-based pain classifiers with pain score zero as the reference in different settings.

	**CNN model**	**Fully connected layers**	**Pain score** **0 vs. 1 vs. 2**	**Pain score** **0 vs. 1/2**	**Pain score** **0 vs. 2**
Accuracy	Resnet34	1 layer (1024, 3)	0.5589	0.7668	0.8593
Sensitivity			0.5589	0.8422	0.8925
F1-score			0.5495	0.7832	0.8638
Accuracy		2 layers	0.6032	0.7711	0.8568
Sensitivity		(1,024, 256)	0.6032	0.8380	0.8514
F1-score		(256, 3)	0.5969	0.7855	0.8561
Accuracy	VGG16	1 layer (1024, 3)	0.5914	0.7578	0.8557
Sensitivity			0.5914	0.6665	0.8499
F1-score			0.5867	0.7141	0.8548
Accuracy		2 layers	0.5871	0.7578	0.8276
Sensitivity		(1,024, 256)	0.5871	0.6908	0.8064
F1-score		(256, 3)	0.5811	0.7405	0.8239
Accuracy	InceptionV1	1 layer (1024, 3)	0.5872	0.7055	0.8302
Sensitivity			0.5872	0.8216	0.8782
F1-score			0.5788	0.7362	0.8380
Accuracy		2 layers	0.5567	0.7587	0.8035
Sensitivity		(1,024, 256)	0.5567	0.8159	0.8338
F1-score		(256, 3)	0.5556	0.7718	0.8093

*CNN, convolutional neural network*.

### Performance of Video-Based Pain Classifiers and the Pain Classifier Without Reference

We then examined the performance of a video-based pain classifier through concatenating vectors of the aforementioned three CNN encoders and BiLSTM with distinct hidden layers (Table 3). We found that the performance of video-based pain classifiers among the polychromous classifier and two dichotomous classifiers was higher than those in the image-based pain classifier. The accuracy in classifying 0 vs. 1/2 was nearly 0.8 and reached ~0.88 to classify 0 vs. 2. Additionally, we further tested the performance of the established classifier without reference, mimicking the clinical scenario in a new patient without an image score of 0 as the reference (Table 4). We found that the performance of both image- and video-based classifiers slightly decreased in classifiers without reference. Notably, the performance of a video-based classifier without reference to differentiate 2 from 0 was up to 0.8906, indicating the established classifier had learned the difference between 0 and 2. Collectively, we established the image and video facial expression-based pain classifier in critically ill patients, with the accuracy to classify 0 vs. 1/2 and 0 vs. 2 were ~0.8 and 0.9, respectively.

**Table 3:** Performance of video-based pain classifiers with different numbers of hidden layers in bidirectional long short-term memory (BiLSTM) networks.

	**Hidden layers**	**Pain score** **0 vs. 1 vs. 2**	**Pain score** **0 vs. 1/2**	**Pain score** **0 vs. 2**
Accuracy	64	0.6144	0.8145	0.8810
Sensitivity		0.6144	0.7947	0.8755
F1-score		0.6123	0.8107	0.8803
Accuracy	128	0.5941	0.8054	0.8461
Sensitivity		0.5942	0.7858	0.7589
F1-score		0.5902	0.8015	0.8314
Accuracy	256	0.6006	0.8268	0.8367
Sensitivity		0.6006	0.8244	0.7500
F1-score		0.5948	0.8264	0.8212

*BiLSTM, bidirectional long short-term memory*.

**Table 4:** Accuracy of proposed image- and video-based pain classifiers with and without reference.

	**Reference**	**Pain score** **0 vs. 1 vs. 2**	**Pain score** **0 vs. 1/2**	**Pain score** **0 vs. 2**
**Image-based pain classifiers**				
Accuracy	Pain score 0	0.6347	0.8000	0.8937
Sensitivity		0.6347	0.8022	0.8826
F1-score		0.6321	0.8004	0.8953
Accuracy	No reference	0.6421	0.7954	0.8771
Sensitivity		0.6421	0.7974	0.9074
F1-score		0.6371	0.7947	0.8724
**Video-based pain classifiers**				
Accuracy	Pain score 0	0.6144	0.8268	0.8810
Sensitivity		0.6144	0.8244	0.8755
F1-score		0.6123	0.8264	0.8803
Accuracy	No reference	0.6130	0.7858	0.8906
Sensitivity		0.6130	0.8016	0.8344
F1-score		0.6102	0.7892	0.8841

## Discussion

In this prospective study, we developed a protocol to obtain video clips of facial expressions in critically ill patients and employed the deep learning-based approach to establish the facial expression-based pain classifier. We focused on the area nearby eyebrow that is less likely to be masked by medical devices and employed an ensemble of three CNN models, such as Resnet34, VGG16, and InceptionV1, to learn pain-associated facial features and BiLSTM for temporal relation between video frames. The accuracy of the dichotomous classifier to differentiate tense/grimacing (1/2) from relaxed (0) facial expression was ~80%, and the accuracy to detect grimacing (2) was nearly 90%. The present study demonstrates the practical application of deep learning-based automated pain assessment in ICU, and the findings shed light on the application of medical artificial intelligence (AI) not only to improve patient care, but also to relieve healthcare workers from the routine workload.

Pain is the fifth vital sign in hospitalized patients but is somehow difficult to assess in critically ill patients who cannot self-report the pain (21, 22). Facial expressions of pain consist of coordinated pain-indicative muscle movements, particularly the contraction of muscles surrounding the eyes, i.e., orbicularis oculi muscle (23). Notably, facial pain responses appear to be consistent across distinct types of pain stimulation, such as pressure, temperature, electrical current, and ischemia (23, 24). A number of studies have explored the physiological basis of how pain signaling leads to pain-indicative muscle movement. Kuramoto et al. recently used facial myogenic potential topography in 18 healthy adult participants to investigate the facial myogenic potential and subsequent facial expressions (25). Furthermore, Kunz used functional MRI (fMRI) to address the association between brain responses in areas that processed the sensory dimension of pain and activation of the orbicularis oculi muscle (26). Although promising, monitoring of facial myogenic potential might be infeasible in critically ill patients given that contact device-associated issues regarding infection control and the potential interference with critical care (27). The possibility of application of fMRI in ICU appears to be low; therefore, using a portable camera to take high-quality video ~1–2 m from the patient as well as AI-based image analyses focusing on eyebrow area as we have shown in the present study has high applicative value in critically ill patients.

It is estimated that more than 50% of patients in ICU experienced experience moderate to severe pain at rest, and 80% of critically ill patients experience pain during procedures (28, 29). Therefore, CPOT, as well as Behavioral Pain Scale (BPS), has been introduced for pain assessment in patients at ICUs in the past two decades, and facial expression is the fundamental domain in both BPS and CPOT given that muscle tension in facial areas, particularly facial area nearby eyebrow, can be directly observed by the caring staff without contact (3, 30). Notably, contactless monitoring in ICU is of increasing importance in the post-coronavirus disease (COVID) era (27). A number of AI-based tools, such as the dynamic relationship of facial landmarks or CNN-learned facial features, have been developed to assess pain in non-ICU patients (7, 23, 31). Nevertheless, the subtle pain-associated movement of facial muscles/landmarks in the non-ICU patient is largely distinct from those in critically ill patients under sedation. Given that patients in ICU often received mechanical ventilation, experienced fear were deprived of normal sleep, felt isolation; therefore, appropriate sedation, at least light sedation, is recommended as a standard of care in critically ill patients and hence leads to difficulties to identify pain based on facial expressions (32). In addition to the impact of sedation on pain assessment, subtle facial muscle movements might also be confounded by facial oedema resulting from fluid overload, which is highly prevalent in critically ill patients who underwent fluid resuscitation, as we have shown in our previous studies (33, 34). Collectively, automated pain assessment based on facial expressions in critically ill patients is currently an unmet need in the research field of medical AI due to the aforementioned difficulties.

Intriguingly, we found a suboptimal performance in the polychromous classifier, whereas the performance in dichotomous classifiers was high. We postulated that the relatively little difference between pain grades 1 and 2 may lead to the reduced performance to differentiate between 1 and 2, and the performance of dichotomous classifiers was high due to the apparent difference between 0 and 1/2. We found a higher performance in video classifiers than those in image classifiers, and this finding indicates that the temporal relation among image frames is crucial to classify pain by facial expressions. A similar finding has been found in pain classifiers using the UNBC-McMaster shoulder pain database (7–9). The accuracy of the leave-one-subject-out 25-fold cross in facial expression-based pain classifier by machine learning approach was ~0.861 using the UNBC-McMaster database (7). Similar to our approach, Rodriguez et al. used VGG to learn basic facial features as well as LSTM to exploit the temporal relation between video frames and reported a further increased accuracy (0.933) in the aforementioned UNBC-McMaster database (8). Similarly, Huang *et al*. proposed an end-to-end hybrid network to extract multidimensional features including time-frame features from images of the UNBC-McMaster database and also found an improved performance (9). Recently, Semwal and Londhe further used distinct fusion network architectures, including CNN-based fusion network to learn both the spatial appearance and shape-based descriptors, as well as decision-level fusion network to learn the domain-specific spatial appearance and complementary features, to improve the performance of pain intensity assessment, with the F1 score, was ~0.94 (10, 11). This evidence highlights the potential application of automated pain assessment based on facial expressions in hospital.

The inevitable medical devices and high heterogeneity in critically ill patients have led to technical difficulties as we have shown in this study. We choose to crop the facial area nearby the eyebrow area, and this approach not only keeps the essential area to detect painful facial expressions but also is essential to extend the established model to clinical scenarios with distinct facial masks, such as the increasing prevalence of wearing a facial mask in the post-COVID era. Moreover, we used a pain score of 0 to train the pain classifiers in this study and further tested the performance of established classifiers without reference (Table 4). Notably, the performance of dichotomous classifiers, particularly the 0 vs. 2 classifier, remains high without reference, indicating that the established model has learned the pain-associated facial expression in critically ill patients.

Timely detection of severe pain, such as pain score 2, is crucial in critical care. Frequent pain assessment is substantial for the identification of the existence of pain and the adjustment dosage of pharmacological analgesic agents or the intensity of non-pharmacological management (1). The previous studies have shown that regular pain assessment is associated with a better outcome, such as ventilator-day, in critically ill patients (35, 36). Severe pain may reflect not only inadequate pain control, but also the potential deterioration of critical illness. For example, increasing pain has been implicated with anxiety, delirium, and poor both short-term and long-term outcomes in critically ill patients (37). Therefore, the automated AI-based pain assessment, particularly timely identification of severe pain/pain score 2, should serve as an actionable AI target, i.e., the detection of pain score 2 indicates the need for immediate evaluation and management by the healthcare worker. Additionally, we have established the user interface to guide the user with regard to quality of the image and the real-time classification of pain based on facial expressions, and the application of the established model should hence reach level 5 of technology readiness level (TRL) (Supplementary Demonstration Video 1) (38, 39).

There are limitations in this study. First, this study is a single center study. However, the pain relevant management in the study hospital is in accordance with the guideline; therefore, the generalization issue should be at least partly mitigated. Second, we recorded the video for 90 s in each record, and a longer duration could further improve the accuracy. Third, we focused on the facial expression in the present study, and more sensors for the other domains of CPOT/BPS are warranted in the future.

## Conclusion

Autonomous facial expression-based pain assessment is an essential issue in critical care but is somehow difficult in critically ill patients due to inevitable masked areas by medical devices and relatively subtle muscle movement resulting from sedation/oedema. In the present prospective study, we established the deep learning-based pain classifier based on facial expression focusing on the area nearby eyebrow, with the accuracy to detect tense/grimacing and grimacing were ~80 and 90%, respectively. These findings indicate a real-world application of AI-based pain assessment based on the facial expression in ICU, and more studies are warranted to validate the performance of the automated pain assessment tool.

## Data Availability Statement

The original contributions presented in the study are included in the article/Supplementary Material, further inquiries can be directed to the corresponding author/s.

## Ethics Statement

The study was approved by the Institutional Review Board of Taichung Veterans General Hospital (TCVGH: CE20325A). The patients/participants provided their written informed consent to participate in this study.

## Author Contributions

C-LW, S-JS, S-FY, C-CC, and W-CC: study concept and design. S-FL, S-JS, and H-JC: acquisition of data. T-LY, C-HC, S-FY, Y-SL, C-CC, and W-CC: analysis and interpretation of data. C-LW and W-CC: drafting the manuscript.

## Funding

This study was supported by and Ministry of Science and Technology Taiwan (MOST 109-2321-B-075A-002). The funders had no role in the study design, data collection and analysis, decision to publish, or preparation of the manuscript.

## Conflict of Interest

The authors declare that the research was conducted in the absence of any commercial or financial relationships that could be construed as a potential conflict of interest.

## Publisher's Note

All claims expressed in this article are solely those of the authors and do not necessarily represent those of their affiliated organizations, or those of the publisher, the editors and the reviewers. Any product that may be evaluated in this article, or claim that may be made by its manufacturer, is not guaranteed or endorsed by the publisher.
