# Supplementary material for: Deep Learning-Based Pain Classifier Based on the Facial Expression in Critically Ill Patients
Source: Front Med (Lausanne). 2022 Mar 17;9:851690. doi: 10.3389/fmed.2022.851690 (PMC8968070; doi:10.3389/fmed.2022.851690)
Supplement: Supplementary file 2 [file Presentation_1.PPTX]

## Slide 1
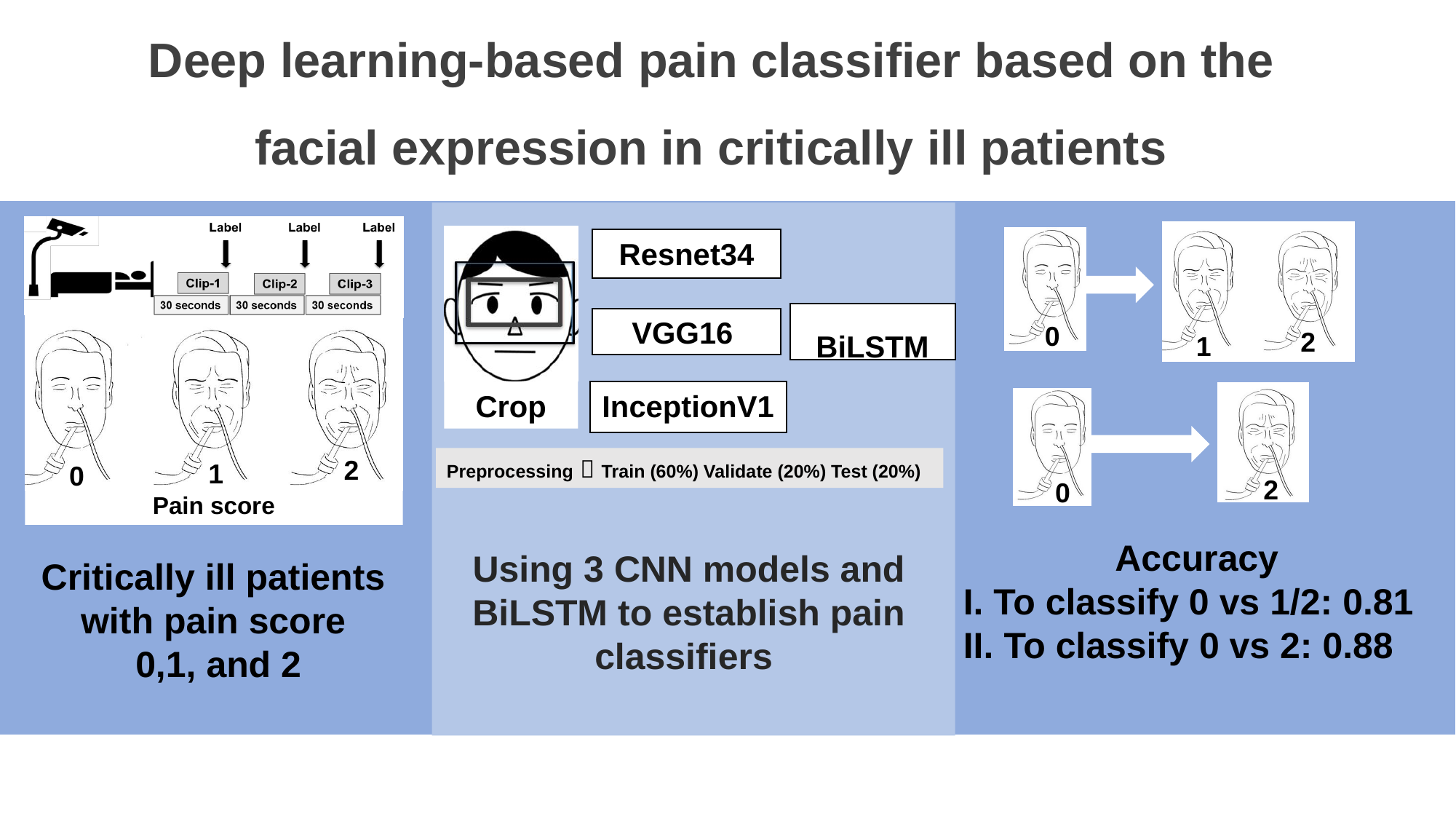

Deep learning-based pain classifier based on the facial expression in critically ill patients
Pain score
Crop
Resnet34
BiLSTM
VGG16
0
2
1
InceptionV1
2
Preprocessing  Train (60%) Validate (20%) Test (20%)
1
0
2
0
Accuracy
I. To classify 0 vs 1/2: 0.81
II. To classify 0 vs 2: 0.88
Using 3 CNN models and BiLSTM to establish pain classifiers
Critically ill patients
with pain score
0,1, and 2
